# Does balneotherapy provide additive effects to physical therapy in patients with subacute supraspinatus tendinopathy? A randomized, controlled, single-blind study

**DOI:** 10.1007/s00484-020-02032-6

**Published:** 2020-10-22

**Authors:** Cihan Koç, Emine Eda Kurt, Fatmanur Aybala Koçak, Hatice Rana Erdem, Naime Meriç Konar

**Affiliations:** 1 Bahçe Physical Therapy and Rehabilitation Hospital, Osmaniye, Turkey; 2 Ahi Evran University Faculty of Medicine, Kırşehir, Turkey

**Keywords:** Subacute supraspinatus tendinopathy, Balneotherapy, Rotator cuff lesions, Physical therapy

## Abstract

This study assessed the additional contribution of balneotherapy on physical therapy in subacute supraspinatus tendinopathy. Ninety patients with subacute supraspinatus tendinopathy were included. They were randomized into two equal groups. In group 1 (*n* = 45), transcutaneous electrical nerve stimulation (TENS), hot pack, ultrasound treatments, and Codman’s and range of motion (ROM) exercises were performed. In group 2 (*n* = 45), balneotherapy was added to the treatment program. In both groups, shoulder active ROM and handgrip strength were measured. Pain was evaluated using a Visual Analogue Scale (VAS) (rest, sleep, movement); functional assessment and quality of life were measured respectively with the Shortened Disabilities of the Arm, Shoulder and Hand Questionnaire (QuickDASH), and the Short Form-36 health survey (SF 36) form. All measurements were repeated before and after 15 treatment sessions. There were statistically significant differences between the before and after assessment parameters in group 1 (all *p* < 0.05), but not for SF-36 General Health Perceptions, SF-36 Mental Health sub-parameters, and handgrip strengths. However, there were statistically significant differences between all the evaluation before and after the treatment in group 2 (all *p* < 0.05). When the two groups were compared in terms of alpha gains, statistically significant differences were observed in favor of group 2 in all measurements (all *p* < 0.05) except for SF-36 Emotional Role Difficulty and SF-36 Mental Health sub-parameters. This study shows that the addition of balneotherapy to physical therapy for subacute supraspinatus tendinopathy can make additional contributions to shoulder ROM, pain, handgrip strength, functional status, and quality of life.

## Introduction

The shoulder joint has the greatest range of movement in the body. Shoulder pain is the third most common problem in the general population, after back and neck pain among musculoskeletal system issues (Roe et al. 2013). Its prevalence varies between 7 and 26%. The wide range of prevalence rates has been explained in the literature by the use of different definitions (Luime et al. 2009). Acute shoulder pain is defined as symptoms lasting up to 6 weeks, subacute lasts 6 to 12 weeks, and chronic pain is defined as symptoms lasting longer than 12 weeks. Studies indicate that the duration of symptoms is the most important in terms of prognosis. Chronic pain makes treatment difficult and increases treatment costs (Reilingh et al. 2008). Periarticular causes account for up to 90–95% of shoulder pain. Among these, rotator cuff lesions are the most common cause. Rotator cuff lesions vary in a broad spectrum from tendinitis to partial and complete tears and calcific tendinopathy. Studies using diagnostic imaging methods in shoulder pain showed that rotator cuff pathologies were most frequently observed in the supraspinatus tendon (Vecchio et al. 1995; Karel et al. 2017).

In treatment, conservative methods such as analgesic and anti-inflammatory drugs, various injections, exercises, and physical therapy are used. Various trends and modalities, hot-cold treatments, deep heating agents, mobilization, and manipulation techniques are used in physical therapy. In cases where conservative treatments are inadequate, surgical methods are used (Filiz and Çakır 2014).

Balneotherapy is frequently used for musculoskeletal diseases including shoulder diseases in our country and some European and Asian countries (Forestier et al. 2017). There are many studies assessing the effects of balneotherapy in hand and knee osteoarthritis, chronic low back pain, and degenerative diseases such as lumbar spondylosis and mechanical neck pain, and in fibromyalgia (Nasermoaddeli and Kagamimori 2005; Fioravanti et al. 2014; Roques and Queneau 2016; Branco et al. 2016).

The additional contribution of balneotherapy to treatment in shoulder pathologies has been investigated in a limited number of studies. However, these studies included patients who were treated with broad definitions such as subacromial impingement syndrome or chronic shoulder pain (Şen et al. 2010; Chary-Valckenhaere et al. 2012). Also, only a few studies indicated the efficacy of balneotherapy in chronic shoulder pain (Şen et al. 2010; Chary-Valckenhaere et al. 2012; Tefner et al. 2015). Only one study showed the beneficial effects of peloid treatment in subacromial impingement syndrome, which is one of the causes of shoulder pain (Şen et al. 2010). The effects of thermal water baths have not been studied adequately in shoulder pathologies.

In light of all these data, the additive effects of balneotherapy on physical therapy in patients with subacute supraspinatus tendinopathy (6–12 weeks) were aimed to investigate. We evaluated health-related quality of life, emotional mood, sleep, pain scores, functional evaluation of the shoulder, handgrip strength, and active range of motion (ROM).

## Material and methods

This single-blind, randomized controlled trial was conducted in the Physical Medicine and Rehabilitation Department Outpatient Clinic of the Ahi Evran University Medical Faculty. Declaration of Helsinki protocols were followed, and local ethics committee approval for the study was obtained (process no: 2018-06/62). The study was performed between March 29, 2019, and April 30, 2019 (ACTRN12619000045112). This study also conforms to all consort guide lines and reports the required information accordingly. The patients were evaluated by a single researcher (CK) both before and after the treatment periods. The researcher was blinded as to which treatment protocol the patients had been ordered.

The G-power (v.3.1.9.2) program was used to determine the sample size, and it was concluded that a minimum of 45 people in each group was required to achieve an effect size of approximately *d* = 0.5 (medium-level effect size) at 80% power and 5% significance level (Cohen 2013).

Patients between the ages of 20–65 with 6–12 weeks of unilateral shoulder pain were examined. Neer, Hawkins, and painful arc tests are provocative tests for subacromial impingement. All tests were performed to the patients. Patients who were positive in at least one of these three tests were evaluated. To be diagnosed with subacute supraspinatus tendinopathy (Burbank et al. 2008), pain severity (VAS 4 and above) is moderate or severe, full passive range of motion were assigned as inclusion criteria.

Other shoulder evaluation tests (Cools et al. 2008), physical examination, laboratory, and diagnostic imaging were performed. Patients with the differential diagnosis of shoulder pain were excluded and patients with subacute supraspinatus tendinopathy with the affected shoulder MRI were included in the study.

In the Neer test, one hand stabilizes the patient’s scapula while the other hand raises the arm into full flexion; a positive test is indicated by pain. The Hawkins test involves flexing the shoulder to 90° then forcibly internally rotating it, though gentle internal rotation has also been recommended. Pain in the shoulder area indicates that the test is positive. When performing the painful arc test, the patient is asked to actively lift the arm in the scapular plane, then slowly reverse the movement. The test is noted positive if the patient has pain between 60–120 degrees of during elevation (Çaliş et al. 2000). A detailed history was taken from all patients. Musculoskeletal system and neurologic examinations were performed and radiologic (shoulder anteroposterior (AP)/lateral, cervical AP/lateral), serologic (acute phase reactants, erythrocyte sedimentation rates, C-reactive protein (CRP), rheumatoid factor (RF)), and biochemical analysis (liver function tests, fasting blood glucose (FBG), urea, uric acid, creatinine) and hemograms were obtained. Magnetic resonance (MR) imaging was performed on the affected shoulder in all cases.

The exclusion criteria were specified as follows: shoulder instability; those who underwent shoulder surgery; positive drop arm test; diagnosed adhesive capsulitis; rotator cuff tear; osteonecrosis; cuff arthropathy or arthritis; a history of shoulder injection in the past one year; acromioclavicular joint pathology; those who received physical therapy and/or received therapeutic balneotherapy in the past one year; those with a history of fracture or dislocation in the shoulder area; calcific tendinitis on radiography; neurologic deficit; regional diseases (cervical radiculopathy, brachial neuritis, complex regional pain syndrome, peripheral neuropathy), rheumatologic, oncologic, infectious disease, coagulopathy, and severe cardiovascular and pulmonary disease; patients with visceral-induced shoulder pain; a history of severe psychiatric illness; and breastfeeding or pregnant women.

**Fig. 1:**
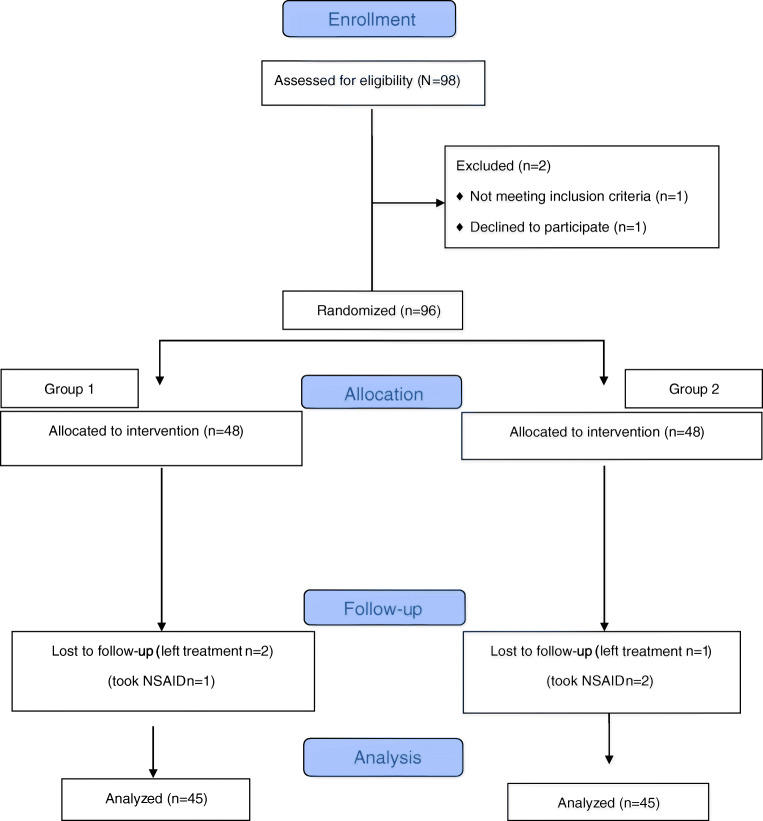
Flow diagram of the study population

According to the inclusion and exclusion criteria, 98 patients who were diagnosed as having subacute supraspinatus tendinopathy were included in the study; eight patients dropped out for various reasons (Fig. 1). The study was completed with 90 patients (53 women and 37 men). The participants were given detailed information about the study and their written approval was obtained.

Patients were randomly divided into two equal groups using the covariate adaptive randomization method (variables: age, sex, education level) with a computer program (Kang et al. 2008).

Group 1 received transcutaneous electrical nerve stimulation (TENS), hot pack, ultrasound (US), and exercise treatment. Group 2 received balneotherapy in addition to the treatments given to group 1. All treatments were performed in a total of 15 sessions; five days per week.

TENS treatment was performed by crossing the electrodes, including the supraspinatus muscle and the aching area, at 60–80 Hz frequency, 100 msec pulse intervals, with a current intensity at 1 to 100 mA, where the patient feels a slight tingling, without causing contraction. During treatment, the patient’s arm was supported with a pillow in a resting position while the patient was sitting. Each session lasted 20 min.

Hot pack treatment was performed by placing a hot pack containing silica gel on the aching shoulder, which was heated in water at 72–75 °C in a boiler, and wrapped in two layers of towels. The treatment was performed once a day for 20 min.

The US treatment was performed by moving the probe with continuous contact in circular movements over the aching shoulder, at a dose of 1.5w/cm^2^ in the continuous mode, for 6 min per day.

For exercises, Codman’s pendulum exercises were given to both groups of patients. The exercises were actively performed by the patient under the supervision and directions of the researcher for 15 min.

In group 2, balneotherapy was given at Kırşehir Terme Spas, which operate under the Department of Physical Medicine and Rehabilitation in Ahi Evran University. The hot mineral water at 42 ± 1 °C contains 98.3 mg/L sulfur, 556 mg/L bicarbonate, 186.7 mg/L sodium, 34.5 mg/L magnesium, 226 mg/L calcium, 232 mg/L chloride, 2.6 mg/L fluoride, and 58.43 mg/L silicate acid. Spa treatment was given to the patients as a whole-body bath and assigned as 20 min.

No analgesic or anti-inflammatory drugs were allowed to be taken during the study. None of the patients were using pregabalin and/or gabapentin.

The demographic and affected shoulders characteristics were recorded. The active ROM (flexion, extension, abduction, internal and external rotation) of the affected shoulder was measured using a goniometer. Grip strength evaluations were performed using a Jamar hand dynamometer. The patients were asked to grade their pain during sleep, rest, and movement using a Visual Analogue Scale (VAS) scoring system. A shortened Disabilities of the Arm, Shoulder and Hand Questionnaire (QuickDASH) and the Short Form-36 health survey (SF-36) quality of life scale were administered before and after the study.

The VAS is a scale used for the evaluation of pain severity. The scale is a 10-cm line with the left-most part showing no pain, and the right-most part showing maximum pain. All patients were asked to mark the most appropriate statement on the line according to the pain (Hong 2011). The SF-36 scale is used measure of quality of life, that consists of 36 items evaluating physical functioning; physical role functioning; emotional role functioning; social role functioning; general health, mental health; bodily pain, and vitality. Scores for the eight domains are calculated by summing up the item scores. Each domain is scored from 0 to 100, with 0 indicating the worst health status and 100 indicating the best health status. The validity and reliability studies of the scale have been performed in the Turkish population (Kocyigit et al. 1999).

QuickDASH is an 11-item questionnaire, that measure physical function and symptoms in patients with upper limb musculoskeletal disorders. The 11 items of QuickDASH handle daily activities, house/garden work, shopping, recreation, self-care, eating, sleep, friends, work, pain, and tingling/numbness. The validity and reliability studies of the scale have been performed in the Turkish population (Düger et al. 2006).

The SPSS 22.0 (IBM Corp. released 2013; IBM SPSS Statistics for Windows, version 22.0. Armonk, NY: IBM Corp.) package program was used for all analyses. The normality of measured data distributions was evaluated using the Shapiro-Wilk test. Continuous data are showed as mean ± standard deviation (SD), and categorical data are presented as percentages (%). If the data were normally distributed, Student’s *t* test was used, and the Mann-Whitney U test was used if the data were not normally distributed. Qualitative comparisons of the groups were performed using the Chi-square test. Additionally paired *t* tests were used to compare repeated measures for each group if the data were normally distributed. And the Wilcoxon test was used if the data were not normally distributed. The threshold for statistical significance was set at *p* < 0.05.

## Results

There was no statistically significant difference between the treatment groups regarding demographic characteristics such as age, sex, body mass index, and education (*p* > 0.05). Also, there was no significant difference in baseline pain duration in either group (*p* > 0.05) (Table 1).

**Table 1:** Demographic characteristics

Variable			Group 1 (*n* = 45)		Group 2 (*n* = 45)		*p* value
			Mean ± SD	Median(min-max)	Mean ± SD	Median(min-max)	
Demographic characteristics	Age		48.771 ± 9.64	52 (20–63)	47.33 ± 7.87	50 (21–59)	0.366^§^
	BMI		28.978 ± 5.23	27.7 (21.6–48.10)	29.56 ± 4.17	29.4 (21–41.10)	0.605^§^
	Duration of pain		7.812 ± 2.32	8 (6–12) weeks	8.16 ± 2.61	9 (6–12) weeks	0.657^μ^
	Sex	Female	30 (66.7%)		23 (51.1%)		0.198
		Male	15 (33.3%)		22 (48.9%)		
	Education	No	11 (24.4%)		8 (17.8%)		0.207
		Primary school	15 (33.3%)		25 (55.6%)		
		High School	14 (31.1%)		9 (20%)		
		University	5 (11.1%)		3 (6.7%)		

^§^*t* test in independent groups

^μ^Mann-Whitney *U* test

Group 1 patients receiving physical therapy and exercises for treatment

Group 2 patients receiving physical therapy, balneotherapy, and exercises for treatment

*BMI* body mass index/kg/m^2^

Positivity-negativity ratios of diagnostic tests such as Neer, Hawkins, and the painful arc test were similar in both treatment groups (all *p* > 0.05) (Table 2).

**Table 2:** Special tests used for diagnosis

Special tests		Group 1 (*n* = 45)	Group 2 (*n* = 45)	*p* value
Neer	Positive	35 (77.8%)	36 (80%)	0.999
	Negative	10 (22.2%)	9 (20%)	
Hawkins	Positive	19 (42.2%)	18 (40%)	0.999
	Negative	26 (57.8%)	27 (60%)	
Painful arc	Positive	40 (88.9%)	41 (91.1%)	0.999
	Negative	5 (11.1%)	4 (8.9%)	
	Negative	30 (66.7%)	30 (66.7%)	

Group 1 patients receiving physical therapy and exercises for treatment

Group 2 patients receiving physical therapy, balneotherapy, and exercises for treatment

In group 1 and group 2, the pre and post-treatment ROM measurements (flexion, extension, internal rotation, and external rotation) were evaluated, and statistical significantly improvements were detected in both groups after the treatments (*p* < 0.05) (Table 3).

**Table 3:** Comparison of pre- and post-treatment range of motion in groups 1 and 2

Group	ROM	Pre-treatment		Post-treatment		*p* value
		Mean ± SD	Median(min-max)	Mean ± SD	Median(min-max)	
Group 1	Flexion	169.56 ± 18.82	180 (100–180)	176.22 ± 8.06	180 (150–180)	0.001^♈^
	Abduction	168.22 ± 21.88	180 (90–180)	174.56 ± 11.96	180 (120–180)	0.001^♈^
	Internal rotation	83.78 ± 12.30	90 (40–90)	87.67 ± 6.87	90 (50–90)	0.007^♈^
	External rotation	72.33 ± 21.34	80 (10–90)	78.78 ± 18.56	90 (20–90)	<0.001^♈^
Group 2	Flexion	162.67 ± 20.38	180 (100–180)	176.11 ± 12.10	180 (110–180)	<0.001^♈^
	Abduction	158.89 ± 25.69	170 (90–180)	174.56 ± 18.02	180 (80–180)	<0.001^♈^
	Internal rotation	75.78 ± 18.40	90 (30–90)	87.56 ± 6.10	90 (60–90)	<0.001^♈^
	External rotation	66.11 ± 20.22	70 (10–90)	81.22 ± 11.78	90 (50–90)	<0.001^♈^

^♈^Wilcoxon test

Group 1 patients receiving physical therapy and exercises for treatment

Group 2 patients receiving physical therapy, balneotherapy, and exercises for treatment

*ROM* range of motion

Additionally, both groups were improved significantly with respect to VAS values (resting, sleep, movement), and QuickDASH scores after the treatments (Table 4) (*p* < 0.05).

**Table 4:** Comparison of pre- and post-treatment VAS, QuickDASH, and Jamar measurements in groups 1 and 2

Group		Pre-treatment		Post-treatment		*p* value
		Mean ± SD	Median(min-max)	Mean ± SD	Median(min-max)	
Group1	Resting VAS	2.78 ± 2.60	2 (0–8)	1.78 ± 2.28	0 (0–8)	<0.001^♈^
	Sleep VAS	7.49 ± 1.78	8 (5–10)	4.67 ± 2.88	5 (0–10)	<0.001^♈^
	Movement VAS	6.95 ± 2.50	7 (0–10)	4.47 ± 2.63	5 (0–10)	<0.001^π^
	QuickDASH	42.34 ± 19.85	40 (6.8–88.6)	27.61 ± 20.14	25 (2.2–86.3)	<0.001 ^π^
	Handgrip strength (Jamar)	24 ± 10.04	23 (8–48)	23.82 ± 11.87	24 (9–50)	0.142 ^π^
Group2	Resting VAS	3.11 ± 2.69	3 (0–8)	0.93 ± 1.37	0 (0–5)	<0.001^♈^
	Sleep VAS	8.2 ± 1.89	8 (4–10)	4.22 ± 2.70	4 (0–10)	<0.001^♈^
	Movement VAS	7.6 ± 2.31	7.5 (0–10)	4.04 ± 2.64	4 (0–10)	<0.001^♈^
	QuickDASH	43.84 ± 21.95	40.45 (6.8–88.6)	19.44 ± 16.48	13.6 (2.2–65.9)	<0.001^♈^
	Handgrip strength (Jamar)	24.71 ± 10.07	22 (4–58)	26.47 ± 12.05	24 (10–60)	<0.001^♈^

^π^Paired *t* test

^♈^Wilcoxon test

Group 1 patients receiving physical therapy and exercises for treatment

Group 2 patients receiving physical therapy, balneotherapy, and exercises for treatment

*VAS* Visual Analogue Scale

*QuickDASH* Quick Disabilities of the Arm, Shoulder and Hand

In the post-treatment Jamar hand dynamometer measurements in group 1, there was no significant difference compared with the pre-treatment measurements (*p* > 0.05). However, the post-treatment measurements of the Jamar hand dynamometer were detected significantly higher than the pre-treatment measurements in group 2 (*p* < 0.05) (Table 4).

In group 1, there were no significant differences between measurements of before and after treatment related with SF 36 General Health, and SF 36 Mental Health values (all *p* > 0.05). Group 2 statistically significant improvements were found in all other sub-parameters (*p* < 0.05). However, there were significant improvements in the post-treatment values in all parameters of SF-36 compared with pre-treatment in group 2 (*p* < 0.05) (Table 5).

**Table 5:** Comparison of pre- and post-treatment SF-36 assessment in groups 1 and 2

Group		Pre-treatment		Post-treatment		*p* value
		Mean ± SD	Median (min-max)	Mean ± SD	Median (min-max)	
Group 1	SF36 Vitality	55.11 ± 21.83	55 (5–95)	57.44 ± 21.26	55 (20–95)	0.012^♈^
	SF36 Physical Function	60.04 ± 23.52	72.2 (11.1–100)	69.59 ± 25.1	72.2 (11–100)	<0.001^♈^
	SF36 Pain	39.39 ± 23.53	42.5 (0–90)	62.39 ± 26.62	67.5 (0–100)	<0.001 ^π^
	SF36 General Health Perception	47.89 ± 24.20	50 (0–100)	48.44 ± 23.42	50 (0–90)	0.498 ^π^
	SF36 Physical Role Limitation	25.22 ± 41.52	0 (0–100)	45 ± 45.73	25 (0–100)	0.002^♈^
	SF36 Emotional Role Limitation	65.92 ± 46.32	100 (0–100)	75.55 ± 41.07	100 (0–100)	0.018^♈^
	SF36 Social Function	80.56 ± 27.25	100 (0–100)	85.28 ± 21.03	100 (37.5–100)	0.024^♈^
	SF36 Mental Health	64.10 ± 18.12	64 (20–96)	65.22 ± 18.5	68 (20–96)	0.136^♈^
Group 2	SF36 Vitality	52.56 ± 23.64	50 (5–95)	66.78 ± 20.15	75 (25–90)	<0.001^♈^
	SF36 Physical Function	57.24 ± 27.65	61.1 (5.6–100)	77.63 ± 20.71	83.3 (22.2–100)	<0.001 ^π^
	SF36 Pain	33.28 ± 21.92	35 (0–90)	67.89 ± 25.28	77.5 (0–100)	<0.001^♈^
	SF36 General Health Perception	47.56 ± 26.15	50 (0–100)	54.33 ± 22.85	55 (10–100)	<0.001 ^π^
	SF36 Physical Role Limitation	29.44 ± 41.73	0 (0–100)	70 ± 42.51	100 (0–100)	<0.001^♈^
	SF36 Emotional Role Limitation	61.48 ± 48.70	100 (0–100)	88.89 ± 31.78	100 (0–100)	<0.001^♈^
	SF36 Social Function	77.78 ± 30.84	100 (0–100)	95.56 ± 10.37	100 (50–100)	<0.001^♈^
	SF36 Mental Health	56.53 ± 21.48	62 (8–96)	68.10 ± 1610	72 (28–88)	<0.001^♈^

^π^Paired *t* test

^♈^Wilcoxon test

Group 1 patients receiving physical therapy and exercises for treatment

Group 2 patients receiving physical therapy, balneotherapy, and exercises for treatment

*SF36* Short Form-36

The difference between the treatment efficacy of the two groups was evaluated using delta gains. There was no significant difference between the two groups regarding SF-36 Emotional and SF36 Mental Health gains (*p* > 0.05). In terms of the delta gains of all other variables, a statistically significant difference was found in favor of group 2 (*p* < 0.05) (Table 6).

**Table 6:** Comparison of Δ (post-treatment–pre-treatment) gains per group

	Treatment group				*p* value
	Group 1		Group 2		
	Mean ± SD	Median(min-max)	Mean ± SD	Median(min-max)	
Flexion	6.67 ± 15.37	0 (− 10, 70)	13.44 ± 17.57	10 (0–70)	0.021^μ^
Abduction	6.33 ± 12.99	0 (− 10, 60)	15.67 ± 21.73	10 (− 10, 80)	0.018^μ^
Internal rotation	3.10 ± 10.05	0 (0–50)	11.78 ± 17.62	0 (− 20, 60)	0.008^μ^
External rotation	6.44 ± 10.20	0 (− 20, 40)	15.11 ± 16.74	10 (− 20, 70)	0.01^μ^
Resting VAS	− 1 ± 1.58	0 (− 6, 1)	− 2.18 ± 2.15	− 2 (− 6, 0)	0.008^μ^
Sleep VAS	− 2.82 ± 2.50	− 2 (− 10, 0)	− 3.98 ± 2.70	− 4 (− 10, 0)	0.034^μ^
Movement VAS	− 2.50 ± 2.46	− 2 (− 9, 2)	− 3.56 ± 2.29	− 4 (− 10, 0)	0.028^μ^
QuickDASH	− 14.73 ± 13.16	− 11.4 (− 51, 3.7)	− 24.4 ± 17.84	− 15.9 (− 75, − 2.2)	0.007^μ^
SF36 Vitality	2.33 ± 9.80	0 (− 45, 20)	14.22 ± 21.56	5 (− 20, 65)	0.028^μ^
SF36 Physical Function	9.55 ± 10.92	11.1 (− 11.1, 44.4)	20.38 ± 18.95	16.6 (0, 61.1)	0.001^§^
SF36 Pain	23 ± 23.79	22.5 (− 42.5, 67.5)	34.61 ± 23.85	32.5 (0, 90)	0.043^μ^
SF36 General Health	0.51 ± 5.46	0 (− 15, 15)	6.78 ± 18.16	0 (− 30, 65)	0.026^μ^
SF36 Physical Role Limitation	19.78 ± 37.69	0 (− 50, 100)	40.56 ± 47.76	25 (− 100, 100)	0.01^μ^
SF36 Emotional Role Limitation	9.63 ± 25.25	0 (0, 100)	27.41 ± 44.54	0 (0–100)	0.068^μ^
SF36 Social Function	4.72 ± 14.42	0 (− 12.5, 75)	17.78 ± 25.35	0 (0–75)	0.003^μ^
SF36 Mental Health	1.13 ± 10.06	0 (− 35, 24)	11.56 ± 19.22	0 (− 8, 60)	0.081^μ^
Hand grip strength (Jamar)	0.71 ± 3.19	0 (− 8, 10)	1.68 ± 3.28	2 (− 3, 13)	0.002^μ^

Δ gain: Post-treatment–pre-treatment measurement

^§^*t* test in independent groups

^μ^Mann-Whitney *U* test

Group 1 patients receiving physical therapy and exercises for treatment

Group 2 patients receiving physical therapy, balneotherapy, and exercises for treatment

*VAS* Visual Analogue Scale

*QuickDASH* Quick Disabilities of the Arm, Shoulder and Hand

*SF36* Short Form-36

## Discussion

In our study, a significant improvement was observed in active ROM measurements, QuickDASH, and VAS (during rest, sleep, movement) scores in both groups (*p* < 0.05). However, the difference in the group receiving additional balneotherapy was significantly higher than in the other group (*p* < 0.05). Similar to our results, in the study of Şen et al. (2010), peloid treatment, which is a method of balneotherapy, provided an increase in shoulder ROM measurements, shoulder function, and a significant improvement in VAS scores. In a multicenter study in which the effectiveness of balneotherapy in shoulder pain associated with chronic cuff tendinopathy was evaluated, a significant improvement was observed in the DASH scores of the group receiving the spa treatment (Chary-Valckenhaere et al. 2012). Although similar results were obtained in the study of Tefner et al. (2015), in which thermal water and balneotherapy was used in patients with chronic shoulder pain, as in our study, no significant difference was found between the groups’ ROM measurements. This result was considered to be caused by the capsular tension and adhesions associated with chronic pathologies of the patients included in the study.

There are many studies on the use of balneotherapy in various musculoskeletal diseases in the literature (Odabaşı et al. 2002; Şen et al. 2007; Herisson et al. 2014). Although still not among the recommended treatment methods in some international treatment guidelines and meta-analyses, it is one of the recommendations of the Turkish League Against Rheumatism (TLAR) for the treatment of knee osteoarthritis and ankylosing spondylitis (Bodur et al. 2011; Tuncer et al. 2012). Also, among the non-pharmacologic treatment recommendations of ankylosing spondylitis in the Assessment of Spondyloarthritis International Society (ASAS)/European League Against Rheumatism (EULAR) prepared by van den Berg (2012), balneotherapy is recommended in combination with other non-pharmacologic treatments or alone in addition to pharmacologic treatment.

The thermal, chemical and anti-inflammatory effects of balneotherapy have been stated in numerous studies in the literature (Gálvez et al. 2018; Morer et al. 2017; Cozzi et al. 2018). Also, its benefits on pain and joint stiffness at the cellular-molecular level have been shown. (Kurt et al. 2016; Koczy et al. 2019; Fioravanti et al. 2011), Balneotherapy provides analgesic effects by preventing the stimulation of nociceptive receptors, reducing pain transmission through the gate control theory of pain stimulating thick nerve-fibers, removing oxygen radicals, and increasing beta-endorphin levels in particular (Yurtkuran et al. 1993; Koczy et al. 2019; Tishler et al. 2004; Bender et al. 2005; Hizmetli and Hayta 2011). Again in the literature, it has been shown that balneotherapy treatments decrease inflammation and, ultimately, pain by increasing anti-inflammatory cytokines (Shehata et al. 2006). In our study, we think that the greater improvement in active joint ROM, pain, and shoulder functions of the group receiving balneotherapy may be related to the pathophysiological mechanisms demonstrated in the studies mentioned above. However, real-life data and studies on patients with shoulder pain are less than the other pain syndrome groups (Karagulle et al. 2017).

There was statistically significant increase in handgrip strength measurements with the Jamar hand dynamometer in the post-treatment results of group 2 compared with pre-treatment (*p* < 0.05). In the analysis of delta gains, the gain in the group receiving balneotherapy was also significantly higher. The pain and inflammation reduction and thermal effect mechanisms of balneotherapy have been investigated in many studies in the literature. In response to heat, the elasticity of tissues containing collagen increases, muscle spasm decreases (possibly reducing pain), and joint function improves (Tishler et al. 2004; Bender et al. 2005; Shehata et al. 2006; Fioravanti et al. 2011). Handgrip strength is a clinical measurement that is aimed to be improved with decreasing pain and spasm.

There were significant improvements in sub-parameters of SF-36, except for general health perception and mental health (*p* < 0.05) in group 1. However, in group 2, there was a significant improvement in all parameters (*p* < 0.05). When the post-treatment changes of the two groups were compared, the well-being was higher in the group receiving balneotherapy, except for the role limitation and mental health sub-parameters due to emotional problems.

Balneotherapy has been shown to increase physical and mental quality of life, reduce anxiety and depression, as well as reduce pain and improve functions (Evcik et al. 2002; Fioravanti et al. 2012; Tefner et al. 2015). These effects are estimated to be due to adaptive modifications, particularly in autonomic and behavioral changes in regulatory systems (Bender et al. 2005). For these reasons, balneotherapy is widely used today for therapeutic purposes. In the study of Çağlar (2015), in which the additional contributions of balneotherapy to physical therapy in various musculoskeletal diseases were investigated, a higher rate of improvement was found in favor of the group that received balneotherapy in all sub-parameters of the quality of life scales. Also, similar results have been revealed concerning quality of life in balneotherapy studies with regional diseases such as knee osteoarthritis, hand osteoarthritis, chronic low back pain, and hip osteoarthritis (Guillemin et al. 1994; Horvath et al. 2012; Kesiktaş et al. 2012; Kovacs et al. 2012; Onat et al. 2014, Kovacs et al. 2016). However, different results were reported in SF-36 sub-parameters in a two-center study examining balneotherapy in chronic shoulder pain, in which the control group was given TENS and exercise (Tefner et al. 2015). There were improvements in both groups in the role limitation related to physical problems, vitality, and pain sub-parameters, but the group receiving balneotherapy had no superiority. Additionally, the role limitations due to emotional problems sub-parameter did not improve in either group.

In the abovementioned studies and in our study, most of the SF-36 sub-parameters improved with the spa treatment in general, but different results were obtained in some sub-parameters. In our study, patients undergoing balneotherapy received daily outpatient treatment due to their clinical conditions and intensity. This situation resulted in patients not being able to benefit from recreational factors that increase quality of life, such as environmental change, stress relief, lifestyle change, and rest, which are thought to contribute to the effectiveness of balneotherapy. Therefore, differences in sub-parameters of the quality of life scale (SF-36) may be related to this fact.

In some of the studies performed with diagnostic framing in which the additional contribution of balneotherapy to treatment methods was evaluated, follow-up that could provide data on long-term permanent effects was conducted. However, in our study, the data only included results of the short-term effects because many of the patients were not present in the long-term follow-up. These outcomes revealed additional contributions of balneotherapy in the early period.

In two studies, although patients with newly diagnosed shoulder pain received primary therapy, it was reported that 40–50% of patients continued to have pain even after 6–12 months (Croft et al. 1996, Winters et al. 1999). Kujipar et al. also indicated, when shoulder pain is taken into account, 80% of the expenditures are made up of patients who do not receive good results despite conservative or surgical treatments (Kuijpers et al. 2006). Balneotherapy is a cost-effective treatment and helps to reduce both the loss of labor force and treatment costs (Van Tubergen et al. 2002). When used together with routine physical therapy methods, balneotherapy can contribute to the treatment of musculoskeletal diseases, especially in early stages, to prevent the symptoms from becoming chronic.

As in our study, the efficacy of balneotherapy should be investigated in specific pathologies, with more extensive series and longer follow-up. Our study is important in terms of being the first on a specific pathology among studies investigating the effectiveness of balneotherapy in shoulder diseases. We believe that this study may be a guide for further research.
